# Expression of *APOBEC3B* mRNA in Primary Breast Cancer of Japanese Women

**DOI:** 10.1371/journal.pone.0168090

**Published:** 2016-12-15

**Authors:** Eriko Tokunaga, Nami Yamashita, Kimihiro Tanaka, Yuka Inoue, Sayuri Akiyoshi, Hiroshi Saeki, Eiji Oki, Hiroyuki Kitao, Yoshihiko Maehara

**Affiliations:** 1 Department of Surgery and Science, Graduate School of Medical Sciences, Kyushu University, Maidashi, Higashi-ku, Fukuoka, Japan; 2 Department of Molecular Oncology, Graduate School of Medical Sciences, Kyushu University, Maidashi, Higashi-ku, Fukuoka, Japan; National Cancer Institute, UNITED STATES

## Abstract

Recent studies have identified the apolipoprotein B mRNA-editing enzyme catalytic polypeptide-like 3B (APOBEC3B) as a source of mutations in various malignancies. APOBEC3B is overexpressed in several human cancer types, including breast cancer. In this study, we analyzed *APOBEC3B* mRNA expression in 305 primary breast cancers of Japanese women using quantitative reverse transcription-PCR, and investigated the relationships between the *APOBEC3B* mRNA expression and clinicopathological characteristics, prognosis, and *TP53* mutations. The expression of *APOBEC3B* mRNA was detected in 277 tumors and not detected in 28 tumors. High *APOBEC3B* mRNA expression was significantly correlated with ER- and PR-negativity, high grade and high Ki67 index. The *APOBEC3B* mRNA expression was highest in the triple-negative and lowest in the hormone receptor-positive/HER2-negative subtypes. The *TP53* gene was more frequently mutated in the tumors with high *APOBEC3B* mRNA expression. High *APOBEC3B* mRNA expression was significantly associated with poor recurrence-free survival in all cases and the ER-positive cases. These findings were almost consistent with the previous reports from the Western countries. In conclusion, high *APOBEC3B* mRNA expression was related to the aggressive phenotypes of breast cancer, high frequency of *TP53* mutation and poor prognosis, especially in ER-positive tumors.

## Introduction

Breast cancer is a heterogeneous disease both pathologically and genetically [1, 2]. Apolipoprotein B mRNA-editing enzyme, catalytic polypeptide-like 3B (APOBEC3B) is a member of a APOBEC3 family of DNA cytosine deaminases [3]. It normally functions in the innate immune responses that protect against retrovirus and retrotransposon propagation. However, this enzyme can also deaminate cytosines in the host genome and generate C-to-T mutations. Recent studies have revealed that APOBEC3B is a significant contributor to the somatic mutation burden among several cancer types [4–7]. APOBEC3B expression is up-regulated in various malignant tumors, including breast cancer, and high APOBEC3B expression is correlated with the presence of certain somatic mutations, particularly in *TP53* [4, 5, 8–10]. High APOBEC3B expression has been associated with aggressive clinicopathological characteristics and was significantly associated with a poor prognosis in untreated ER-positive (ER+) patients as well as in ER+ patients receiving adjuvant endocrine therapy [11]. In ER+/lymph-node-negative cases, APOBEC3B expression was an independent poor prognostic factor for disease-free survival (DFS) [11]. Another recent study using two large datasets also revealed that APOBEC3B expression is associated with aggressive and proliferative features. Elevated APOBEC3B expression was associated with recurrence in the luminal tumor subtype [10]. Thus, APOBEC3B is a marker of poor prognosis for ER+ breast cancer, which strongly suggests that genetic aberrations induced by APOBEC3B contribute to breast cancer progression or resistance to treatment [10, 11].

On the other hand, a germ-line deletion polymorphism in *APOBEC3B*, which leads to no expression of APOBEC3B, has been associated with an increased risk of breast cancer [12, 13]. Homozygosity for the APOBEC3B null allele is associated with a higher incidence of breast cancer [14]. The frequencies of this deletion differ by ethnicity: 1% in Africans, 6% in Europeans, 37% in Asians and 93% in Oceanians. [15]. Homozygous deletion of *APOBEC3B* did not affect the tumor aggressiveness or the prognosis [10].

In the present study, we evaluated the expression of *APOBEC3B* mRNA and investigated the relationships between the *APOBEC3B* mRNA expression and the clinicopathological features, the frequency of the mutation in the *TP53* gene, and the prognosis in Japanese women with breast cancer.

## Materials and Methods

### Patient population and tumor specimens

The institutional review board of Kyushu University approved this study. A total of 894 primary breast cancer patients underwent surgery without neoadjuvant systemic therapy in the Department of Surgery and Science, Kyushu University, between 1994 and 2012. Of these, 305 cases for which archival tissue samples for an immunohistochemical analysis and/or extraction of the genomic DNA and total RNA were available were included in this study. Immediately after surgery, the specimens for extraction of the genomic DNA and total RNA were placed in liquid nitrogen and stored at –80°C. Written informed consent was obtained from all of the patients in this study. The clinical data were obtained from the patients’ medical records. The AJCC/UICC TNM classification and stage groupings were used. The estrogen receptor (ER), progesterone receptor (PR), HER2 status, and Ki67 labeling index were evaluated as described previously [16].

### *APOBEC3B* mRNA expression

APOBEC3B expression was analyzed using quantitative reverse transcription-polymerase chain reaction (qRT-PCR). Total RNA was extracted from the frozen specimens with the TRIZOL reagent (Invitrogen Japan K.K., Tokyo, Japan) in accordance with the manufacturer’s recommendations. The extracted RNA was pretreated with RNase-free DNase, and the cDNA was then synthesized with random hexamer primers and Superscript III reverse transcriptase (Invitrogen Japan K.K.) in accordance with the manufacturer’s instructions. The *APOBEC3B* mRNA expression was normalized to that of the constitutive housekeeping gene *TATA binding protein (TBP)* [17]. The primers and probe were Hs00358981-m1 for APOBEC3B and Hs00427620-m1 for TBP (Applied Biosystems, Waltham, MA, USA).

### PCR direct sequencing of the *TP53* gene and single-nucleotide polymorphism- comparative genomic hybridization

Genomic DNA was extracted as described previously [18, 19]. PCR direct sequencing was performed for exon 5–9 of the *TP53* gene as previously described [18]. The presence of genetic alterations was investigated by single-nucleotide polymorphism- comparative genomic hybridization (SNP-CGH, HumanOmni1-Quad BeadChip; Illumina Inc.), and the copy number variation was analyzed with the software program GenomeStudio V2009.1 (Illumina Inc.) as described previously [18, 20].

### Statistical analyses

The statistical analyses were performed using the JMP software package, version 9.0.2 (SAS Institute Inc., Cary, NC, USA). The associations between the *APOBEC3B* mRNA expression and clinicopathological characteristics were assessed using χ^2^ tests. The relapse-free survival (RFS) was defined as the time from surgery to the first breast cancer event, including loco-regional recurrence, distant metastasis, or a new cancer in the contralateral breast. Survival curves were plotted using the Kaplan–Meier method and the association between survival and each variable was determined by the log-rank test. For multivariate analysis of the survival data, Cox proportional hazards model was used. Differences were considered to be significant at p < 0.05.

## Results

### *APOBEC3B* mRNA expression

Of the 305 cases evaluated, expression of *APOBEC3B* mRNA was not detected in 28 tumors, which was defined as “no” *APOBEC3B* mRNA expression. Among 277 tumors, in which *APOBEC3B* mRNA expression was detected, *APOBEC3B* mRNA expression was dichotomized based on the median value as either “low” or “high” expression. The associations between the *APOBEC3B* mRNA expression and the clinicopathological characteristics were compared among three groups: no, low, or high *APOBEC3B* mRNA expression (Table 1).

**Table 1:** The association between APOBEC3B mRNA expression and clinicopathological characteristcs.

Factors	none (n = 28)	APOBEC3B mRNA expression		*P-*value
		low (n = 139)	high (n = 138)	
Age (yo) (mean ± SE)	53.2 ± 2.5	56.4 ± 1.1	55.5 ± 1.1	0.4863
Nodal status				
Negative	21 (75.0)	89 (64.0)	85 (61.6)	0.3869
Positive	7 (25.0)	50 (36.0)	53 (38.4)	
pathological tumor size (pT)				
0	0 (0)	5 (2.9)	5 (2.9)	0.8032
1	10 (42.9)	58 (38.4)	51 (36.0)	
2	15 (50.0)	65 (46.4)	71 (45.6)	
3	3 (7.1)	11 (10.1)	11 (14.0)	
Histology				
DCIS	0(0)	5 (3.6)	5 (3.6)	0.2574
Invasive ductal ca	25 (89.3)	125 (89.9)	123 (89.1)	
Invasive lobular ca	1 (3.6)	4 (2.9)	4 (2.9)	
Metaplastic ca	0 (0)	1 (0.7)	5 (3.6)	
Others	2 (7.1)	4 (2.9)	1 (0.7)	
Nuclear grade				
1	7 (26.9)	75 (55.6)	24 (18.6)	<0.001
2	6 (23.1)	24 (17.8)	32 (24.8)	
3	13 (50.0)	36 (26.7)	73 (56.6)	
Ki67 labeling index (mean ± SE)				
	21.9 ± 4.7	20.2 ± 2.3	33.1 ± 2.3	0.0002
ER				
Negative	5 (17.9)	20 (14.4)	60 (43.5)	<0.001
Positive	23 (82.1)	119 (85.6)	78 (56.5)	
PR				
Negative	8 (28.6)	31 (22.3)	70 (51.1)	<0.001
Positive	20 (71.4)	108 (77.7)	67 (48.9)	
HER2				
Negative	17 (60.7)	116 (86.6)	95 (72.0)	0.002
Positive	10 (35.7)	18 (13.4)	37 (28.0)	
N.D.	1 (3.6)	0 (0)	0 (0)	
Subtype				
HR+/HER2-	14 (50.0)	104 (77.6)	63 (47.7)	<0.001
HR+/HER2+	8 (28.6)	11 (8.2)	15 (11.4)	
HR-/HER+	2 (7.1)	7 (5.2)	22 (16.7)	
Triple negative	3 (10.7)	12 (9.0)	32 (24.2)	
N.D.	1 (3.6)	0 (0)	0 (0)	
			(%)	

pT; pathological tumor size, HR; horomne receptor, ND; not determined

**Fig 1:**
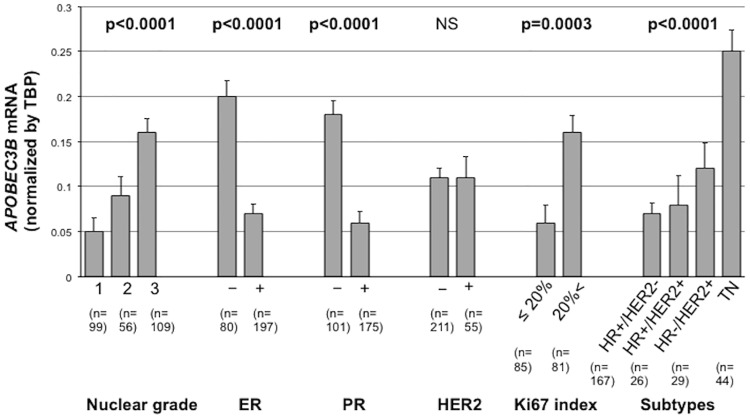
APOBEC3B mRNA expression according to the clinicopathological factors. *APOBEC3B* mRNA expression was significantly higher in higher grade, ER−, PR− and high-Ki67 tumors. *APOBEC3B* mRNA expression was highest in TN tumors. HR; hormone receptor.

High *APOBEC3B* mRNA expression was significantly correlated with higher nuclear grade (p<0.0001), higher Ki67 labeling index (p = 0.0002) and ER and PR negativity (p<0.0001 and p<0.0001). There were no significant associations between the *APOBEC3B* mRNA expression and the age, lymph node metastasis, pathological stage and histology. HER2 positivity was highest in the tumors with no *APOBEC3B* mRNA expression, and lowest in the tumors with low *APOBEC3B* mRNA expression (p = 0.0023). In terms of breast cancer subtype, high *APOBEC3B* mRNA expression was significantly associated with the triple negative (TN) subtype. Low *APOBEC3B* mRNA expression was associated with HR+/HER2- subtype, and no expression was most frequent in HR+/HER2+ subtype (p<0.0001) (Table 1). Intriguingly, the tumors lacking *APOBEC3B* mRNA expression seemed to be more aggressive than those with low *APOBEC3B* mRNA expression, due to higher nuclear grade and higher HER2-positivity. Fig 1 shows the *APOBEC3B* mRNA expression according to the clinicpoathplogical factors. In Fig 1, the relative *APOBEC3B* mRNA expression level normalized by *TBP* mRNA level was shown. The data from tumors without *APOBEC3B* mRNA expression were excluded. Relative *APOBEC3B* mRNA expression were higher in high-nuclear-grade (p<0.0001), ER–(p<0.0001), PR–(p<0.0001) tumors. *APOBEC3B* mRNA expression was also higher in tumors with high Ki67 index (p = 0.0003) and TN tumors (p<0.0001) (Fig 1).

### Association between *APOBEC3B* mRNA expression and the patient prognosis

**Fig 2:**
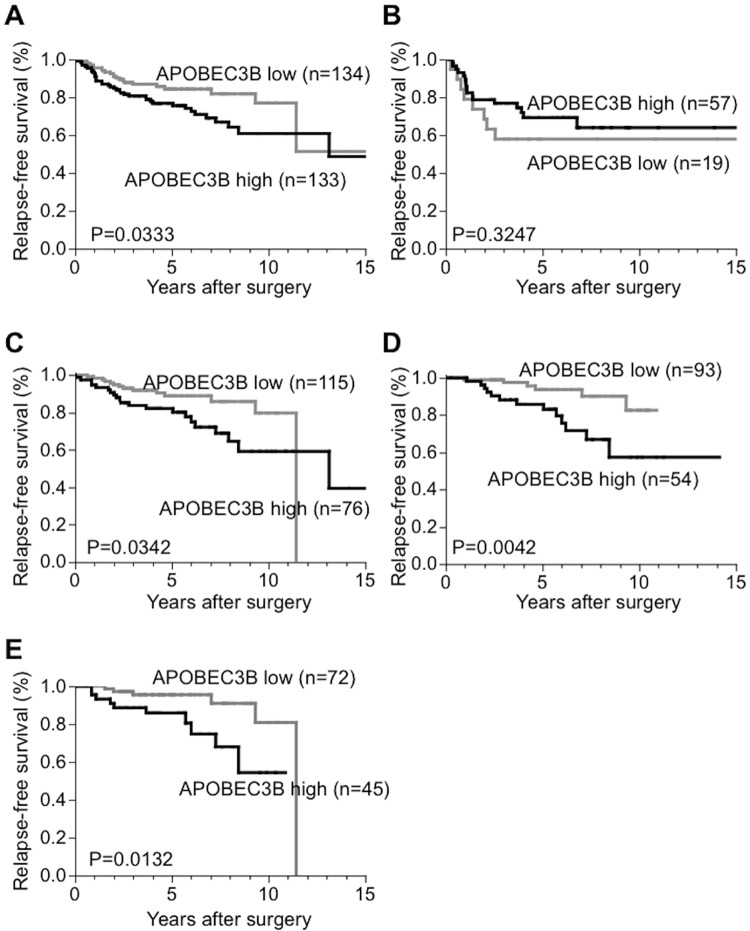
The relationship between APOBEC3B mRNA and the prognosis (relapse-free survival). A. All cases, B. ER− cases, C. ER+ cases, D. ER+ and adjuvant endocrine+ cases, E. ER+ and node-negative cases. High APOBEC3B expression was associated with shorter relapse-free survival in all cases, ER+ cases, ER+ with adjuvant endocrine therapy cases and ER+ and node-negative cases (A, C, D, E).

The association between *APOBEC3B* mRNA expression and the patient prognosis was examined in 295 invasive breast cancer patients.The median follow-up period was 4.93years (range, 0.5–19.9 years). Fig 2 shows the prognosis data of the patients with high and low *APOBEC3B* mRNA expression. As shown in Fig 2, high *APOBEC3B* mRNA expression was associated with shorter RFS compared with than low expression (p = 0.0333, Fig 2A). While *APOBEC3B* mRNA expression was not associated with RFS in ER*–*tumors (Fig 2B), high *APOBEC3B* mRNA expression was significantly associated with shorter RFS in ER+ tumors (p = 0.0342, Fig 2C), especially in the patients who had received adjuvant endocrine therapy (p = 0.0042, Fig 2D) or the node-negative patients (p = 0.0132, Fig 2E). There were no statistically significant differences in the prognosis between high and no *APOBEC3B* mRNA expression, and no marked differences were recognized in the prognosis between low and no *APOBEC3B* mRNA expression (data not shown).

Univariate and multivariate analyses were performed to assess the effect of the clinicopathological factors on the RFS (Table 2). The “no expression cases” were not included in “low expression group”. According to the univariate analysis, high APOBEC3B mRNA expression was significantly associated with poor RFS in all cases and ER+ cases. However, a multivariate Cox hazard analyses analysis showed that high APOBEC3B mRNA was not an independent prognostic factor. When analyzing the ER+ with adjuvant endocrine therapy or LN-negative ER+ cases, high APOBEC3B mRNA expression was significantly associated with poor RFS on both univariate and multivariate analyses (Table 2).

**Table 2:** Univariate and multivariate analysis for recurrence-free survival.

Factors		Univariate analysis			Multivariate analysis		
		HR	95% CI	P value	HR	95% CI	P value
All cases (n = 267)							
Age (y.o.)	≤40 vs. 41≤	1.73	0.32–1.14	0.1074			
Tumor size	T2, T3 vs. T1	2.70	1.56–4.93	0.0003	2.00	1.06–3.96	
LN meta.	positive vs. negative	2.59	1.55–4.41	0.0003	1.94	1.07–3.54	
Nuclear grade	3 vs. 1, 2	2.10	1.22–3.65	0.0076	1.49	0.77–2.89	
ER	positive vs. negative	0.50	0.30–0.84	0.0099	0.97	0.42–2.21	
PR	positive vs. negative	0.40	0.23–0.67	0.0005	0.44	0.20–0.97	
HER2	positive vs. negative	1.98	1.09–3.45	0.0253	1.00	0.50–1.96	
*APOBEC3B* mRNA	high vs. low	1.76	1.05–3.02	0.0324	1.26	0.71–2.31	
ER-negative cases (n = 76)							
Age (y.o.)	≤40 vs. 41≤	1.13	0.27–3.26	0.8480			
Tumor size	T2, T3 vs. T1	3.49	1.47–9.60	0.0037	2.49	1.01–7.03	0.0463
LN meta.	positive vs. negative	4.50	1.97–11.1	0.0003	3.63	1.56–9.19	0.0027
Nuclear grade	3 vs. 1, 2	0.74	0.30–2.06	0.5350			
PR	positive vs. negative	0.55	0.03–2.61	0.5229			
HER2	positive vs. negative	1.27	0.56–2.80	0.5521			
*APOBEC3B* mRNA	high vs. low	0.66	0.29–1.61	0.3420			
ER-positive cases (n = 191)							
Age (y.o.)	≤40 vs. 41≤	2.47	1.08–5.14	0.0329	3.44	1.37–7.94	
Tumor size	T2, T3 vs. T1	2.29	1.13–5.00	0.0207	2.43	1.13–5.69	
LN meta.	positive vs. negative	1.72	0.87–3.44	0.1151			
Nuclear grade	3 vs. 1, 2	2.63	1.24–5.49	0.0127	2.07	0.92–4.30	
PR	positive vs. negative	0.39	0.19–0.90	0.0282	0.31	0.14–0.74	
HER2	positive vs. negative	2.22	0.82–5.12	0.1102			
*APOBEC3B* mRNA	high vs. low	2.07	1.05–4.19	0.0364	1.41	0.67–3.01	
ER-positive with adjuvant endocrine therapy cases (n = 147)							
Age (y.o.)	≤40 vs. 41≤	1.89	0.53–5.27	0.2938			
Tumor size	T2, T3 vs. T1	1.70	0.67–4.86	0.2679			
LN meta.	positive vs. negative	1.13	0.44–2.81	0.7853			
Nuclear grade	3 vs. 1, 2	1.93	0.67–5.04	0.2103			
PR	positive vs. negative	0.23	0.07–0.74	0.0160	0.30	0.11–0.97	0.0440
HER2	positive vs. negative	1.47	0.24–4.47	0.5592			
*APOBEC3B* mRNA	high vs. low	3.73	1.47–10.6	0.0052	3.28	1.27–9.45	0.0138
ER-positive and LN-negative cases (n = 117)							
Age (y.o.)	≤40 vs. 41≤	1.97	0.55–5.81	0.2727			
Tumor size	T2, T3 vs. T1	3.81	1.33–12.4	0.0121	3.40	1.00–13.4	0.0493
Nuclear grade	3 vs. 1, 2	3.32	1.06–10.1	0.0394	1.54	0.44–5.31	0.4875
PR	positive vs. negative	0.69	0.19–4.45	0.6460			
HER2	positive vs. negative	4.08	1.11–12.3	0.0356	2.73	0.63–10.7	0.1672
*APOBEC3B* mRNA	high vs. low	3.57	1.27–11.5	0.0158	4.51	1.31–18.3	0.0169

LN meta.; lymph node metastasis, ER; estrogen receptor, PR; progesterone receptor

### Associations between *APOBEC3B* mRNA expression and *TP53* mutation

We next examined the relationship between *APOBEC3B* mRNA expression and a mutation in the *TP53* gene, exon 5–9. We evaluated the *TP53* sequence in 28 tumors lacking *APOBEC3B* as well as in 60 tumors with low and 60 tumors with high *APOBEC3B* mRNA expression. Any mutations in TP53 gene were evaluated. Confirmation whether it is non-germline SNPs or not was not performed. Mutations in the *TP53* gene were detected in 15 (25%), 7 (11.9%) and 2 (7.1%) tumors with high, low and no *APOBEC3B* mRNA expression, respectively. High *APOBEC3B* mRNA expression was significantly associated with high frequencies of the *TP53* mutation compared with no or low *APOBEC3B* mRNA (p = 0.0312, Table 3).

**Table 3:** The association between APOBEC3B mRNA expression and TP53 gene mutation.

*TP 53*	*APOBEC3B* mRNA		P-value
exon 5–9	none (n = 28) or low (n = 60)	high (n = 60)	
wild-type	78 (88.6)	45 (75.0)	0.0312
mutant	10 (11.4)	15 (25.0)	
		(%)	

### Deletion at the *APOBEC3B* gene locus in the tumor lacking *APOBEC3B* mRNA expression

**Fig 3:**
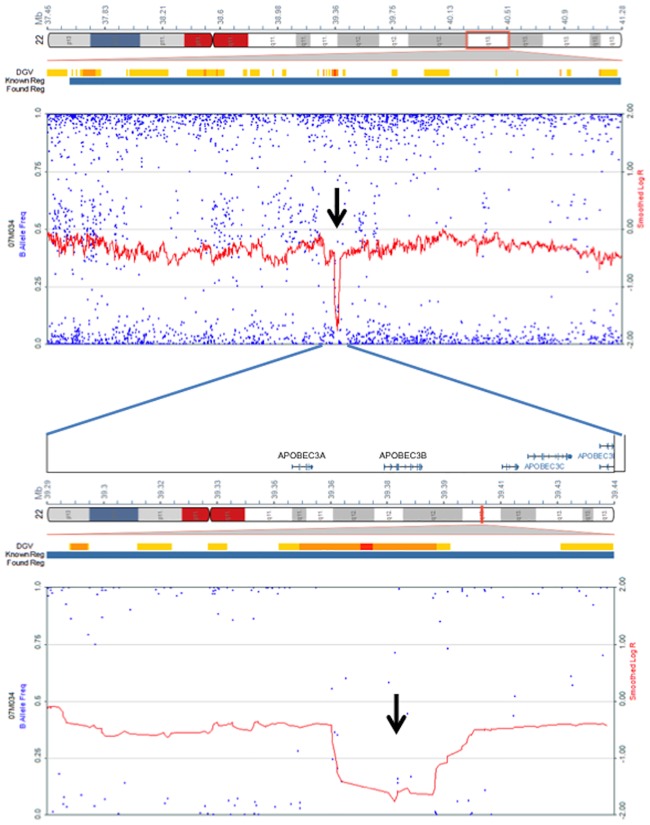
Deletion of APOBEC3B gene locus at chromosome 22 analyzed by SNP-CGH. An apparent deletion was recognized at the APOBEC3B gene locus (arrow). In this case, APOBEC3B mRNA expression was not detected.

SNP-CGH data were available for 26 tumors. Of these, apparent deletion of *APOBEC3B* gene locus was recognized in only one case (Fig 3). In this case, no *APOBEC3B* mRNA expression was detected. The lack of *APOBEC3B* mRNA may have been associated with the deletion of the *APOBEC3B* gene locus in this case.

## Discussion

In this study, we demonstrated that high expression of *APOBEC3B* mRNA was significantly associated with aggressive phenotypes of breast cancer and poor RFS. The poor prognosis of the patients with high-expression tumors are more significant in ER+ tumors, especially in patients who had received adjuvant endocrine therapy and node-negative patinets. High expression of *APOBEC3B* mRNA was also associated with *TP53* gene mutation. We also found that there are some individuals in which *APOBEC3B* mRNA could not be detected, and these tumors are more often HER2-positive, relatively high grade, and have low frequencies of *TP53* mutation, with a deletion of the *APOBEC3B* gene locus observed in one particular case. This is the first study to evaluate the relationship between *APOBEC3B* mRNA expression and the clinicopathological factors, prognosis, and *TP53* mutation status in a large cohort of Japanese breast cancer patients including tumors lacking *APOBEC3B* mRNA expression.

In a previous study conducted in a large cohort of breast cancer cases, the *APOBEC3B* mRNA levels were significantly higher in subjects with a high tumor grade and ER—and PR—tumors [11]. These results are consistent with the data obtained in our study. Sieuwerts et al. showed that high *APOBEC3B* mRNA expression was associated with poor prognosis in ER+ breast cancer, and that high *APOBEC3B* mRNA expression was an independent prognostic factor in ER+, lymph-node-negative patients [11]. In our study, high *APOBEC3B* mRNA was expression significantly associated with poor RFS in all cases as well as ER+ cases, although not to a statistically significant degree in multivariate analyses. However, the analysis for ER+, lymph-node-negative patients revealed that high *APOBEC3B* mRNA expression was an independent poor prognostic factor. There was substantial heterogeneity in our data, probably due to the small sample size. However, the fact that similar results were obtained is very meaningful.

Previous studies have shown the association of APOBEC3B for the elevated levels of DNA damage and mutation in several breast cancer cell lines [4, 5, 21]. APOBEC3B knockdown cells had fewer C-to-T mutations in the *TP53* and *c-Myc* genes than controls [4, 5]. Moreover, in tumors with *TP53* mutations, APOBEC3B expression was elevated [10]. *TP53* mutations are common in breast cancer, and approximately 30% of breast cancers harbor a somatic mutation in the *TP53* gene [9, 22, 23]. Thus, high expression of APOBEC3B might cause *TP53* mutation in breast cancer. In our study, the frequency of the *TP53* gene mutation was also higher in tumors with high *APOBEC3B* mRNA expression than in those with low or lacking *APOBEC3B* mRNA expression. Although the mechanism of the upregulation of *APOBEC3B* mRNA expression has not been been fully elucidated, a recent study showed the induction of APOBEC3B in vitro by environmental factors such as DNA damage and viral infection [24].

It has been shown that APOBEC3B is lacking in some people due to a deletion polymorphism near the *APOBEC3B* gene [14, 25]. There is a large ethnic difference in the frequency of the deletion. The incidence of *APOBEC3B* deletion polymorphism is higher in East-Asian, American and Oceanian populations than in African and European populations [25]. In our study, the tumor in which *APOBEC3B* gene locus was deleted did not express *APOBEC3B* mRNA. Thus, the deletion of *APOBEC3B* gene locus might be a mechanism of lacking *APOBEC3B* mRNA in this tumor.

Tsuboi et al. recently reported on the *APOBEC3B* mRNA expression in Japanese breast cancer patients [26]. In their report, *APOBEC3B* mRNA expression was not related to the frequency of *TP53* mutation or survival time, although the high *APOBEC3B* mRNA expression were associated with subtype, lymph node metastasis and nuclear grade. However, they analyzed only 93 cases, a smaller sample size than ours. Therefore, their results were not conclusive.

Several limitations associated with the present study warrant mention. This study is retrospective, and the sample size is smaller than those of previous reports from European populations. There was also substantial heterogeneity in the background of the patients. However, we were able to demonstrate that high *APOBEC3B* mRNA expression is associated with the aggressiveness of breast cancer and a poor prognosis in ER+ breast cancer, regardless of ethnic difference.

## Conclusions

Our findings here suggest that high *APOBEC3B* mRNA expression is associated with aggressive phenotypes, high frequency of *TP53* mutation, and poor prognosis of breast cancer, especially for ER+ breast cancer.

## References

[1] Stephens PJ, Tarpey PS, Davies H, Van Loo P, Greenman C, Wedge DC, et al The landscape of cancer genes and mutational processes in breast cancer. Nature. 2012;486(7403):400–4. 10.1038/nature11017 22722201 PMC3428862

[2] Cancer Genome Atlas N. Comprehensive molecular portraits of human breast tumours. Nature. 2012;490(7418):61–70. 10.1038/nature11412 23000897 PMC3465532

[3] Refsland EW, Harris RS. The APOBEC3 family of retroelement restriction factors. Curr Top Microbiol Immunol. 2013;371:1–27. 10.1007/978-3-642-37765-5_1 23686230 PMC3934647

[4] Burns MB, Lackey L, Carpenter MA, Rathore A, Land AM, Leonard B, et al APOBEC3B is an enzymatic source of mutation in breast cancer. Nature. 2013;494(7437):366–70. 10.1038/nature11881 23389445 PMC3907282

[5] Burns MB, Temiz NA, Harris RS. Evidence for APOBEC3B mutagenesis in multiple human cancers. Nature genetics. 2013;45(9):977–83. 10.1038/ng.2701 23852168 PMC3902892

[6] Taylor BJ, Nik-Zainal S, Wu YL, Stebbings LA, Raine K, Campbell PJ, et al DNA deaminases induce break-associated mutation showers with implication of APOBEC3B and 3A in breast cancer kataegis. eLife. 2013;2:e00534 10.7554/eLife.00534 23599896 PMC3628087

[7] Lawrence MS, Stojanov P, Polak P, Kryukov GV, Cibulskis K, Sivachenko A, et al Mutational heterogeneity in cancer and the search for new cancer-associated genes. Nature. 2013;499(7457):214–8. 10.1038/nature12213 23770567 PMC3919509

[8] Kuong KJ, Loeb LA. APOBEC3B mutagenesis in cancer. Nature genetics. 2013;45(9):964–5. 10.1038/ng.2736 23985681 PMC3965181

[9] Silwal-Pandit L, Vollan HK, Chin SF, Rueda OM, McKinney S, Osako T, et al TP53 mutation spectrum in breast cancer is subtype specific and has distinct prognostic relevance. Clinical cancer research: an official journal of the American Association for Cancer Research. 2014;20(13):3569–80. 24803582 10.1158/1078-0432.CCR-13-2943

[10] Cescon DW, Haibe-Kains B, Mak TW. APOBEC3B expression in breast cancer reflects cellular proliferation, while a deletion polymorphism is associated with immune activation. Proceedings of the National Academy of Sciences of the United States of America. 2015;112(9):2841–6. 10.1073/pnas.1424869112 25730878 PMC4352793

[11] Sieuwerts AM, Willis S, Burns MB, Look MP, Meijer-Van Gelder ME, Schlicker A, et al Elevated APOBEC3B correlates with poor outcomes for estrogen-receptor-positive breast cancers. Hormones & cancer. 2014;5(6):405–13. 25123150 10.1007/s12672-014-0196-8 PMC4228172

[12] Long J, Delahanty RJ, Li G, Gao YT, Lu W, Cai Q, et al A common deletion in the APOBEC3 genes and breast cancer risk. Journal of the National Cancer Institute. 2013;105(8):573–9. 10.1093/jnci/djt018 23411593 PMC3627644

[13] Xuan D, Li G, Cai Q, Deming-Halverson S, Shrubsole MJ, Shu XO, et al APOBEC3 deletion polymorphism is associated with breast cancer risk among women of European ancestry. Carcinogenesis. 2013;34(10):2240–3. 10.1093/carcin/bgt185 23715497 PMC3786378

[14] Nik-Zainal S, Wedge DC, Alexandrov LB, Petljak M, Butler AP, Bolli N, et al Association of a germline copy number polymorphism of APOBEC3A and APOBEC3B with burden of putative APOBEC-dependent mutations in breast cancer. Nature genetics. 2014;46(5):487–91. 10.1038/ng.2955 24728294 PMC4137149

[15] Henderson S, Fenton T. APOBEC3 genes: retroviral restriction factors to cancer drivers. Trends Mol Med. 2015;21(5):274–84. 10.1016/j.molmed.2015.02.007 25820175

[16] Hisamatsu Y, Tokunaga E, Yamashita N, Akiyoshi S, Okada S, Nakashima Y, et al Impact of FOXA1 expression on the prognosis of patients with hormone receptor-positive breast cancer. Annals of surgical oncology. 2012;19(4):1145–52. 10.1245/s10434-011-2094-4 21984487

[17] Leonard B, Hart SN, Burns MB, Carpenter MA, Temiz NA, Rathore A, et al APOBEC3B upregulation and genomic mutation patterns in serous ovarian carcinoma. Cancer research. 2013;73(24):7222–31. 10.1158/0008-5472.CAN-13-1753 24154874 PMC3867573

[18] Saeki H, Kitao H, Yoshinaga K, Nakanoko T, Kubo N, Kakeji Y, et al Copy-neutral loss of heterozygosity at the p53 locus in carcinogenesis of esophageal squamous cell carcinomas associated with p53 mutations. Clinical cancer research: an official journal of the American Association for Cancer Research. 2011;17(7):1731–40. 21325068 10.1158/1078-0432.CCR-10-1996

[19] Okada S, Tokunaga E, Kitao H, Akiyoshi S, Yamashita N, Saeki H, et al Loss of heterozygosity at BRCA1 locus is significantly associated with aggressiveness and poor prognosis in breast cancer. Annals of surgical oncology. 2012;19(5):1499–507. 10.1245/s10434-011-2166-5 22179631

[20] Peiffer DA, Le JM, Steemers FJ, Chang W, Jenniges T, Garcia F, et al High-resolution genomic profiling of chromosomal aberrations using Infinium whole-genome genotyping. Genome Res. 2006;16(9):1136–48. 10.1101/gr.5402306 16899659 PMC1557768

[21] Harris RS. Molecular mechanism and clinical impact of APOBEC3B-catalyzed mutagenesis in breast cancer. Breast cancer research: BCR. 2015;17:8 10.1186/s13058-014-0498-3 25848704 PMC4303225

[22] Borresen-Dale AL. TP53 and breast cancer. Human mutation. 2003;21(3):292–300. 10.1002/humu.10174 12619115

[23] Olivier M, Langerod A, Carrieri P, Bergh J, Klaar S, Eyfjord J, et al The clinical value of somatic TP53 gene mutations in 1,794 patients with breast cancer. Clinical cancer research: an official journal of the American Association for Cancer Research. 2006;12(4):1157–67. 16489069 10.1158/1078-0432.CCR-05-1029

[24] Middlebrooks CD, Banday AR, Matsuda K, Udquim KI, Onabajo OO, Paquin A, et al Association of germline variants in the APOBEC3 region with cancer risk and enrichment with APOBEC-signature mutations in tumors. Nature genetics. 2016;48(11):1330–8. 10.1038/ng.3670 27643540 PMC6583788

[25] Kidd JM, Newman TL, Tuzun E, Kaul R, Eichler EE. Population stratification of a common APOBEC gene deletion polymorphism. PLoS genetics. 2007;3(4):e63 10.1371/journal.pgen.0030063 17447845 PMC1853121

[26] Tsuboi M, Yamane A, Horiguchi J, Yokobori T, Kawabata-Iwakawa R, Yoshiyama S, et al APOBEC3B high expression status is associated with aggressive phenotype in Japanese breast cancers. Breast Cancer. 2015. 10.1007/s12282-015-0641-8 26476745

